# Single-shot time-folded fluorescence lifetime imaging

**DOI:** 10.1073/pnas.2214617120

**Published:** 2023-04-12

**Authors:** Valentin Kapitany, Vytautas Zickus, Areeba Fatima, Guillem Carles, Daniele Faccio

**Affiliations:** ^a^Extreme Light, School of Physics & Astronomy, University of Glasgow, Glasgow G12 8QQ, UK; ^b^Plasmonics and Nanophotonics Laboratory, Department of Laser Technologies, Center for Physical Sciences and Technology, Vilnius LT-10257, Lithuania

**Keywords:** FLIM, single-shot, time-folded cavity, physics-inspired neural network

## Abstract

Fluorescence lifetime imaging is an important tool in bioimaging that allows one to detect subtle changes in cell dynamics and their environment. Most time-domain approaches currently involve scanning a single illumination point across the sample, which can make imaging dynamic scenes challenging, while single-shot “rapid lifetime determination” can suffer from large uncertainties when the lifetime is not appropriately sampled. Here, we propose a time-folded fluorescence lifetime imaging microscopy (TFFLIM) approach, whereby a time-folding cavity provides multiple spatially sheared replicas of the lifetime, each shifted temporally with respect to a fixed time gate. This provides a robust, single-shot FLIM approach that we experimentally validate across a broad lifetime range on fluorescent beads and Convallaria samples.

## Significance

This paper describes a single-shot fluorescence lifetime imaging (FLIM) method. We use an optical cavity to create temporally delayed and spatially sheared replicas of the fluorescent decay signal onto a time-gated intensified charged-coupled device (iCCD). This modality allows different portions of the decay signal to be sampled in parallel by a single camera, bypassing the convention of scanning a gate. The technique is validated experimentally on fluorescent beads and cells.

Fluorescence lifetime imaging quantifies the time-dependent properties of fluorophores (1–3) and is typically employed for imaging biological phenomena. FLIM measurements are utilized to extract information about the local environment of the fluorophores, such as concentration of oxygen, pH, as well as to reveal protein–protein interactions, changes in membrane tension, etc. (3–7). Despite the fact that FLIM has been recently shown to benefit clinical diagnostics (8–10), wider adoption of the technique in clinical settings is still hindered due to limited field of view (FOV) and slow acquisition speeds of commercial FLIM systems (10). The main bottleneck in conventional systems is the point-scanning image acquisition, which prohibits the acquisition of instantaneous full FOV information. To tackle this, recent innovation has focused on improving the acquisition speeds of confocal scanning systems, in particular with resonant scanning and spinning-disc systems (11) in conjunction with detection approaches based, for example, on photomultiplier tubes (PMT) with time-correlated single photon counting electronics (TCSPC). Nonetheless, if dynamic scenes and large FOVs are desired, or plane-illumination schemes like light-sheet imaging or total internal reflection fluorescence (TIRF) microscopy are used, wide-field systems would be the solution.

Wide-field single-photon avalanche diode (SPAD) arrays have gained popularity both in TCSPC (12) and time-gated operation (13, 14). Wide-field FLIM systems have also been shown that utilize microchannel-plate gated optical intensifiers in addition to arrayed PMT detectors (15) or charge-coupled device (CCD) cameras (16, 17). Such systems benefit from a high-fill factor and low noise and were designed for low-light imaging applications. The fluorescence decay information in such systems is extracted by scanning a gate that samples different temporal sections of the decay. In the extreme case where only 2 gates are used, the acquired data lends itself to relatively fast and simple lifetime recovery techniques referred to as Rapid Lifetime Determination (RLD) (18).

Recently, variations of RLD have been proposed that bypass scanning a gate, including a novel wide-field FLIM system utilizing Pockels cells and a 4f optical cavity (19), which creates a time-integrated measurement in a single snapshot image. The cost of this approach is that 16 replicas of the FOV are imaged onto the detector, therefore potentially limiting total FOV or resolution.

Attempts have been made at using a single, gated camera in combination with temporal streaking of the fluorescent signal in order to determine its lifetime. For example, combining compressed ultrafast photography (CUP) (20) with a dual camera detection scheme has been demonstrated to allow wide-field streaked imaging (21). However, CUP and related approaches rely on streak cameras and leave the need for setups with simpler or cheaper components. Heshmat et al. have also shown that it is possible to retrieve the temporal evolution of systems that evolve at the picosecond-to-nanosecond scale with a time-folding approach whereby multiple time-delayed discrete replicas of a laser pulse result from its periodic optical round trips in an optical cavity (22).

We propose a FLIM that sits in between the discrete sampling of RLD and the continuous streaking of CUP-based approaches. We use a purely optical, time-folded cavity to shear a fluorescent signal in space and time and replicate it within our field of view. This approach retains most of the field of view, while also producing multiple, discrete time-gated samples of the fluorescent signal. These samples act as a series of parallel measurements, similar to taking multiple RLD acquisitions of the same sample with different gate positions at the same time.

Lifetime images are then retrieved by an inverse retrieval scheme and a physics-inspired neural network, for predicting lifetime robustly and quickly, respectively. This approach is validated on various samples over a broad range of lifetimes.

## Results

### Time-Folded Cavity.

**Fig. 1.**
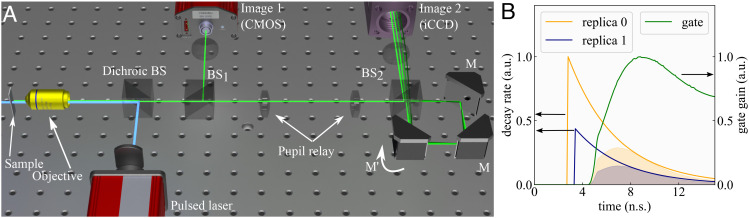
(*A*) The system consists of an objective with epifluorescence illumination, 2 cameras (standard CMOS, and a gated iCCD), and a time-folding optical cavity assembled from a beamsplitter (*BS*′) and 3 mirrors, 1 of which (*M*′) controls the direction of the replicas and their separation in the image plane. A 1:1 telescope relays the pupil of the objective into the cavity to minimize vignetting and cropping of the FOV. (*B*) The optical cavity creates replicas of the original signal with an added time lag, caused by the cavity’s round trip time. Each replica is translated spatially by *M*′, and its amplitude shrinks according to the splitting ratio of *BS*_2_. The iCCD signal measured from each replica is represented by the area of the shaded region under the curves; this area is the dot product of the exponential decay and the gate, shown truncated here for visualization; *SI Appendix* for the full gate shape.

The proposed experimental layout is shown in Fig. 1*A*. A uniform sample of fluorescent molecules will decay according to an exponential probability density function. Our optical cavity replicates this decay signal and retards each replica in time by ∼0.59 ns, as shown in Fig. 1*B*. A tilted mirror *M*′ in the cavity adds an angular offset to the signal as it leaves the cavity. The replicas pass through a lens; hence, this angular offset becomes a lateral translation on the iCCD. Consequently, the iCCD intensifies different portions of the signal on different pixels.

The noise-free time-folded lifetime signal at a pixel *s*_*i*, *j*_ on the iCCD is

[1] $$\begin{array}{cc} s_{i,j}= & \;r_{0}\int G\left(t\right)A_{i,j}e^{\frac{t_{0}-t}{\tau_{i,j}}}H\left(t-t_{0}\right)dt\\ & +r_{1}\int G\left(t\right)A_{i,j-1y}e^{\frac{t_{0}+t_{c}-t}{\tau_{i,j-y}}}H\left(t-t_{0}-t_{c}\right)dt\\ & +...\\ & +r_{N}\int G\left(t\right)A_{i,j-Ny}e^{\frac{t_{0}+Nt_{c}-t}{\tau_{i,j-Ny}}}H\left(t-t_{0}-Nt_{c}\right)dt, \end{array}$$

where *r*_*n*_ for *n* ∈ 0, 1, ..., *N* is a constant, which represents the power transmitted in the n-th round trip through the beamsplitter. The splitting ratio at the excitation wavelength was experimentally measured as 51.5:48.5. *G*(*t*) is the temporal profile of the time-gated intensifier on the iCCD camera. *A* is the amplitude of the decay, and *τ* is the corresponding lifetime. *H*(*t* − *α*) is the Heaviside step function. The *N* terms represent *N* round trips; we determined *N* = 5 to be a good cutoff point, i.e., further round trips had a poor signal-to-noise ratio (SNR) or were not visible. The image shear, measured in pixels per round trip, is y. At each round trip, the replicas are also delayed by the cavity round trip time, *t*_*c*_.

In parallel to the iCCD acquisition, a CMOS camera also acquires an image where each CMOS pixel *i*, *j* records an intensity

[2] $$\begin{array}{c} q_{i,j}=k\int A_{i,j}e^{\frac{t_{0}-t}{\tau_{i,j}}}dt, \end{array}$$

which corresponds to the time-integrated fluorescence recorded at each pixel. Here, *k* is a factor that represents the amplitude ratio of the measurement obtained on the iCCD and CMOS cameras. This arises due to the product of the splitting ratio of *BS*_1_, the pixel sizes, and fill factors of the two cameras, and their respective quantum yields at the given emission wavelength. As such, it is constant over our experiments.

### Uncertainty Analysis.

We assess the viability of our imaging modality by analyzing the lower bound of our prediction uncertainty. This lower bound is found by considering a Bayesian model and by assuming that the noise in the sCMOS is negligible compared to the iCCD. We then calculate the probability of a true sample lifetime *τ* given a lifetime estimate $\hat{\tau }$ obtained from a set of noisy iCCD measurements $\hat{s}$ (i.e., $\hat{s}\left(\tau \right)$):

[3] $$\begin{array}{c} p\left(\tau |\hat{\tau }\right)\equiv p\left(\tau |\hat{s}\right)=\frac{p\left(\hat{s}|\tau \right)p\left(\tau \right)}{p(\hat{s})}. \end{array}$$

This formalism allows us to derive lifetime uncertainty from the well-studied noise properties of an iCCD; *Methods* for details.

**Fig. 2.**
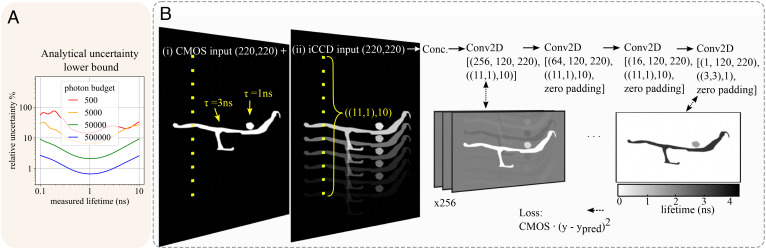
(*A*) We show the lower bound on the relative uncertainty of the lifetime prediction (calculated using Eq. **3**) for photon budgets of 500, 5,000, 50k, and 500k photons (incident on the iCCD). Similarly to RLD (18), our measurements have minimal uncertainty around some lifetime domain, which can be controlled by the position of the gate with respect to the excitation pulse, and the time delay between each sheared image. Our system is optimized to measure ≈ 1 ns signals. (*B*) Outline of the CNN. (*i* and *ii*) The CMOS and iCCD images are shown. They have two lifetime populations: a small sphere with a 1-ns lifetime and a long, thin structure with a 3-ns lifetime. The lifetime of the sample affects the relative intensities of subsequent replicas on the iCCD, with longer lifetime samples losing more intensity between one replica and the next. The dilated Conv2D kernel is shown in yellow; a dilation rate of 10 means that only every 10th kernel value is nonzero, creating a sparse receptive field. The inputs are concatenated, then processed by 3 Conv2D layers with kernel sizes of (11 × 1) and dilation rates of 10. The first hidden layer is shown; it consists of 256 feature maps. Since the inputs are not zero-padded, the size of the feature map decreases compared to the inputs. The output is a lifetime prediction. Training loss is mean squared error weighted by the corresponding intensity values.

We evaluate the uncertainty lower bound of our system for four different example photon budgets in Fig. 2*A*. With 5,000 photons (incident on the iCCD), our lifetime prediction error is less than 10% for lifetimes between 0.36 and 2.6 ns. For a photon budget of 50,000, the same is true for over 2 orders of magnitude of lifetimes, from 0.1 to 10 ns. In the presence of thermal noise or added Poissonian noise on the CMOS, we expect the same overall trend in lifetime uncertainties, at slightly higher values.

### Data Analysis.

We propose two methods to retrieve the lifetime map. One is inverse retrieval (IR) with gradient descent, which is computationally slow but tractable. The other is a physics-inspired convolutional neural network (CNN) trained on simulated data; this method is fast yet intractable.

The IR pipeline optimizes our guess of the lifetime map by iteratively computing the expected iCCD signal for a given lifetime map and calculating the gradient of mean squared error between this expected iCCD signal and the measurement with respect to the lifetime (called the data fidelity term), with gradient descent to update the lifetime guess. This process is analytically tractable. It can however stagnate in local minima of the mean squared error landscape. Therefore, we constrain the IR via an L2-norm regularizer on the lifetime map. Our optimization problem searches for the optimal solution $\hat{\tau }$:

[4] $$\begin{array}{c} \begin{array}{cc} \hat{\tau }= & \;\underset{\tau }{\arg\min}\;C\left(\tau \right)\text{, where}\\ C(\tau )= & \;\|P\left(\tau \right)-\hat{s}{\|}_{2}+\alpha {\left\|\tau \right\|}_{2}^{2}\\ & \;\;\text{subject to}\;\tau \geq 0. \end{array} \end{array}$$

The first term of the cost function *C*(*τ*) represents the data fidelity term, and the second term represents the regularizer. The inverse retrieval is thus a nonlinear ridge regression problem.

Our CNN algorithm, shown in Fig. 2*B*, is trained on a set of synthetic iCCD and CMOS image pairs. It uses 2D convolutional (Conv2D) layers with dilated kernels. Dilation ensures explicit causal dependence of the feature maps on the inputs: the sparse receptive field of the kernel means information is drawn only from pixels with causal dependence in the forward model, minimizing overfitting and matching the physics of the problem. Since the convolutional kernels maintain this dilated shape until the penultimate layer, this causal dependence persists until the deeper layers. The final Conv2D layer’s (3 × 3) kernels mimic sliding window binning, commonly used in lifetime fitting to increase the SNR. Training lifetime labels are in the range of 0.1 to 8 ns. For more details on the IR and the CNN, *Methods*.

We apply both IR and CNN to predict the lifetimes of the center of the field of view. In this region, we have information about all the replicas that enter and leave the field of view, allowing us to predict lifetimes with minimal uncertainty.

### Validation on Beads.

**Fig. 3.**
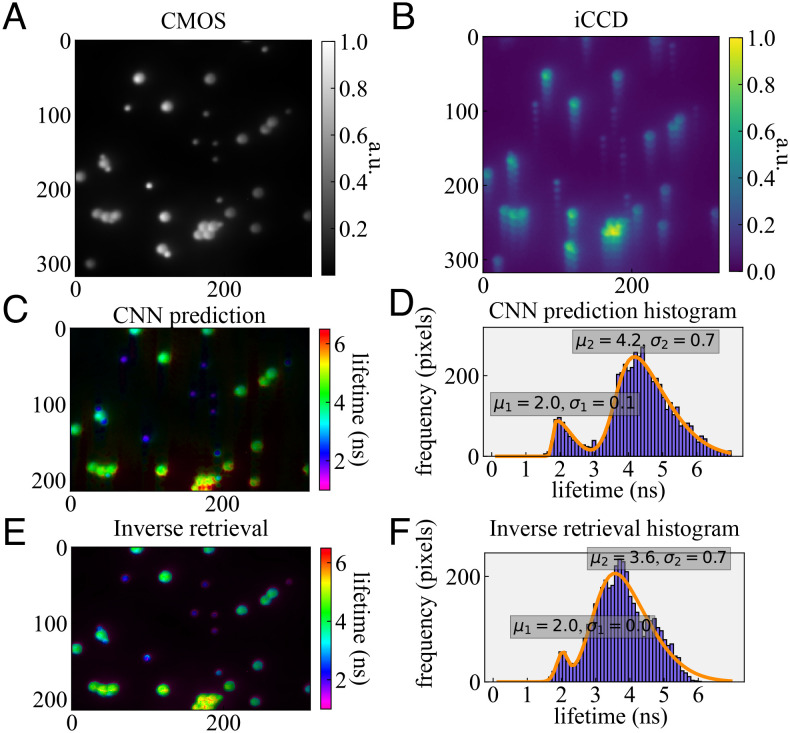
(*A* and *B*) We show a mixture of 2 and 4 μm beads on the CMOS and iCCD. (*C* and *E*) The CNN predicts the two lifetime modes present in the sample clearly. The predicted lifetime distribution is fitted with a bimodal skewed Gaussian, yielding populations of 2.0 ± 0.1 and 4.2 ± 0.7 ns. (*E* and *F*) The IR predicts similar lifetimes, distributed as 2.0 ± 0.0 and 3.6 ± 0.7 ns.

We prepared samples of fluorescent beads of 2 μm and 4 μm diameters with corresponding expected lifetimes of 2.1 ns and 4 ns measured on a separate FLIM system (*Methods* for details). We show results for an FOV containing several such beads in Fig. 3. Exposure times were 1 s.

The 2- and 4-μm beads show bimodal lifetime distributions and are clearly distinguishable by their lifetimes. Moreover, both the neural network and IR yield very similar lifetime distributions, cross-validating one another. We fitted bimodal skewed Gaussian distributions to intensity-weighted (the weighting is used to remove dark pixels) histograms of the pixel values. For the CNN, peak values of 2.0 and 4.2 ns with standard deviations of 0.1 and 0.7 ns were obtained, for the small and large beads, respectively. IR yielded values of 2.0 ± 0.0 and 3.6 ± 0.7 ns, respectively. These values are in good agreement with the expected lifetimes. The prediction uncertainties of the longer lifetime beads are greater than that of the shorter lifetime ones, as expected from Fig. 2*A*.

### Testing on Cells.

**Fig. 4.**
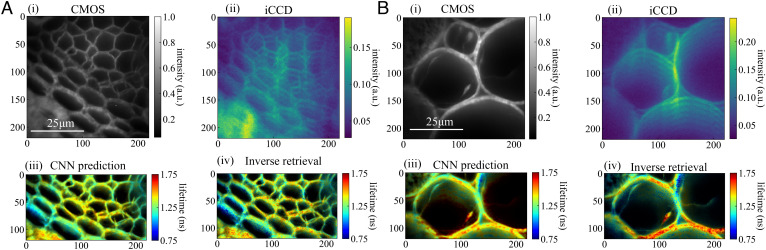
Lifetime map of Convallaria stained with acridine orange. The CNN and inverse retrieval show remarkably similar lifetime patterns, cross-validating one another. The CNN output is a little smoother, while the inverse retrieval has sharper edges but also more granularity; this can be attributed to the final layer of the CNN acting as a form of (learned) sliding window averaging. Reconstructions were intensity-weighted, as the regions in between cell walls expressed low signal. (*A*), (*i* and *ii*) The CMOS and iCCD images of the first region of interest. (*iii* and *iv*) The global lifetime distributions were 1.30 ± 0.09 and 1.29 ± 0.11 ns for the CNN and IR, respectively. (*B*), (*i* and *ii*) CMOS and iCCD images of the second region of interest. (*iii* and *iv*) In the second region of interest, the global lifetime distributions were 1.34 ± 0.11 and 1.30 ± 0.15 ns for the CNN and IR, respectively. We note that the artifacts produced by the inverse retrieval are largely suppressed by the CNN’s final layer, but sharp edges and high-frequency lifetime variations are also smoothed.

We then tested our model on Convallaria cells stained with acridine orange. Mean lifetimes of 1.30 and 1.29 ns for region-of-interest (ROI) 1 (Fig. 4*A*) were obtained with the CNN and IR, respectively. In ROI 2 (Fig. 4*B*), the mean lifetimes predicted by the CNN and IR were 1.34 and 1.30 ns, respectively. These distributions closely match our previously published results of 1.29 ± 0.49 ns (13) on the same sample type. Moreover, the samples do not have a flat lifetime, and the CNN and IR agree on the local lifetime pattern, cross-validating one another. This demonstrates that even though these samples are not spatially sparse like the beads (thus, their time-folded replicas on the iCCD overlap), TFFLIM is capable of predicting lifetimes with good fidelity.

## Discussion

We have demonstrated a method for obtaining fluorescence lifetime images using a time-folded optical cavity in conjunction with an iCCD camera and a CMOS camera. We derive an inverse modeling approach and designed a physics-inspired neural network architecture, thus predicting sample lifetimes for this imaging scheme. TFFLIM samples the signal for different delays, mimicking parallel time-gated measurements, which are acquired in a single snapshot. We validated our approach on beads and tested it on biological samples, obtaining lifetimes in the expected ranges for both. We mathematically demonstrate that the cavity delivers a relatively uniform prediction uncertainty for a wide lifetime range.

The simultaneous spatial and temporal shearing implies that all the information required for retrieving the final lifetime image is acquired in a single measurement. This yields an advantage over scanning time-gated approaches, which sample the signal sequentially for various gate positions. Finally, since we acquire multiple time-gated measurements with different gate positions in parallel, our method could theoretically be expanded to single-shot multiexponential retrieval, analogously to multiexponential RLD (23).

## Materials and Methods

### Setup.

The cavity was assembled from a 50:50 beamsplitter cube (Thorlabs CCM1-BS013/M), two right-angle turning prism mirrors (Thorlabs CCM1-E02/M), an elliptical mirror mounted with a length-wise extended 3D printed mirror mount (Thorlabs H45E1) on a kinematic mount (Thorlabs KM100CP/M) allowing for fine adjustment of replica separation and angle. The imaging optics consisted of a pair of f = 250-mm achromatic doublets (Thorlabs AC254-250-A-ML) which relayed the pupil of a Zeiss 40 × 0.75NA microscope objective. Replicas were imaged onto an iCCD (Andor iStar 334T) with an f = 500-mm achromatic doublet (Thorlabs AC254-500-A-ML).

The replica separation and direction can be found experimentally from the 2D Fourier transform of the iCCD image. The shear direction (and tilt from the desired direction) of the replicas is important as the reconstruction algorithm implicitly assumes that this direction is known. Small deviations from the expected shear direction (even of a few degrees) can lead to artifacts in the form of bands of higher lifetimes that can become very evident for larger angular deviations. The 2D Fourier transform allows us to precisely measure tilt in the shear direction, as well as the replica separation, and subsequently allows the iCCD image to be corrected before processing. In *SI Appendix*, we show examples of sheared images with and without a tilt alongside the relative reconstructions.

A 30:70 beamsplitter cube (Thorlabs BS019) before the pupil relay provided the imaging path for the CMOS camera (Thorlabs CS895MU) with f = 200-mm achromatic doublet (Thorlabs AC254-200-A-ML). A fluorescein isothiocyanate (FITC) filter set was used for fluorescence imaging (Thorlabs MDF-FITC), and Horiba DeltaDiode DD-485L provided the pulsed illumination for fluorescence excitation. The laser diode emits at 470 nm with a 1-MHz repetition rate; the iCCD was triggered at 500 kHz (on every second pulse).

### Samples.

The fluorescent bead samples were prepared by drop-casting isopropanol-diluted bead solution on a microscope slide. Once dried, the beads were washed once with isopropanol to wash away weakly adhered beads. Both 2 μm (Merck, L4530) and 4 μm (Bangs Laboratories Inc., FSDG006) diameter beads were prepared in the same fashion. Acridine orange–stained Convallaria samples were obtained from Johannes Lieder GmbH & Co. KG (Catalog number As3212). In literature, Yellow-Green dye (2 μm Merck beads) has been reported with lifetimes of 2.1 ns (24). Dragon Green dye (4 μm Bangs Laboratory beads) excited at 485 nm has been measured to fluoresce with a 3.4-ns (25) and 4-ns lifetime (26). We validated the lifetime of our samples using a SPAD array (Horiba, FLIMERA); *SI Appendix* for details.

### Inverse Retrieval.

Our forward model is based on Eqs. **1** and **2**. Let us represent as **P** the mathematical function that takes as its input the lifetime map τ and the amplitude values *A* and performs the integration followed by the shifted sum of the replicas as mentioned in Eq. **1**; then, we can concisely write the measurement registered on the iCCD sensor as

[5] $$\begin{array}{c} \hat{s}=P\left(\tau ,A\right)+n, \end{array}$$

where n is measurement noise. Integrating Eq. **2** for *t* from *t*_0_ to ∞ yields *q*_*i*, *j*_ = *kA*_*i*, *j*_*τ*_*i*, *j*_. This can be rearranged as *A*_*i*, *j*_ = $\frac{q_{i,j}}{k\tau_{i,j}}$, i.e., the CMOS measurement is used to evaluate the amplitude of decay *A*_*i*, *j*_, which is then fed to the inverse retrieval algorithm. For this, the factor *k* in Eq. **2** has been calibrated experimentally and is a one-time calibration for the system. The lifetime values, *τ*, thus become the only unknown variables of function **P** henceforth. The cost function we optimize is written in Eq. **4**:

[6] $$\begin{array}{c} \begin{array}{cc} \hat{\tau }= & \;\underset{\tau }{\arg\min}\;C\left(\tau \right)\text{, where}\\ C(\tau )= & \;\|P\left(\tau \right)-\hat{s}{\|}_{2}+\alpha {\left\|\tau \right\|}_{2}^{2}\\ & \;\;\text{subject to}\;\tau \geq 0. \end{array} \end{array}$$

We initiate the optimization with a random guess for the lifetime map τ. The algorithm then uses gradient descent to reach the optimal solution for the variables τ. If the lifetime map at some iteration *n* is represented by *τ*^(*n*)^, then the solution at the next iteration progresses as

[7] $$\begin{array}{c} \begin{array}{c} \tau^{\left(n+1\right)}=\tau^{\left(n\right)}-\beta {\left[\nabla_{\tau }C\left(\tau \right)\right]}_{\tau =\tau^{\left(n\right)}}. \end{array} \end{array}$$

Here, *β* is the step size in the negative gradient direction and is determined in each iteration by backtracking line search (27). ∇_*τ*_*C*(*τ*) is the gradient of the cost function with respect to *τ*:

[8] $$\begin{array}{c} \begin{array}{c} \nabla_{\tau }C\left(\tau \right)=\frac{\left(P\left(\tau \right)-\hat{s}\right)}{\|P\left(\tau \right)-\hat{s}{\|}_{2}}\frac{\partial P(\tau )}{\partial \tau }+2\alpha \sum_{i,j}\tau_{i,j}. \end{array} \end{array}$$

We evaluate the above partial derivative by considering the discrete version of Eq. **1**, giving

[9] $$\begin{array}{c} \begin{array}{cc} \frac{\partial P(\tau )}{\partial \tau }{|}_{i,j}= & -f_{1}\sum_{t}G\left(t\right)q_{i,j}e^{\frac{t_{0}-t}{\tau_{i,j}}}\left[\frac{t_{0}-t}{\tau_{i,j}^{3}}+\frac{1}{\tau_{i,j}^{2}}\right]\\ & -f_{2}\sum_{t}G\left(t\right)q_{i,j-y}e^{\frac{t_{0}+t_{c}-t}{\tau_{i,j-y}}}\left[\frac{t_{0}+t_{c}-t}{\tau_{i,j-y}^{3}}+\frac{1}{\tau_{i,j-y}^{2}}\right]\\ & -...\\ & -f_{6}\sum_{t}G\left(t\right)q_{i,j-5y}e^{\frac{t_{0}+5t_{c}-t}{\tau_{i,j-5y}}}\left[\frac{t_{0}+5t_{c}-t}{\tau_{i,j-5y}^{3}}+\frac{1}{\tau_{i,j-5y}^{2}}\right]. \end{array} \end{array}$$

We use the same notation as in Eqs. **1** and **2**.

### Convolutional Neural Network.

We developed a CNN to retrieve experimental lifetime maps, as an alternative, faster approach to the numerical inversion method described above. The network uses two inputs—the iCCD image and the CMOS image—to predict the lifetime of the sample. We trained the network on a fully synthetic dataset, generated with the forward model in Eq. **5**, with model and noise parameters obtained from experimental data. A set of 10,000 270 × 220 pixel intensity and lifetime seed pairs were used to generate 10,000 220 × 220 single-shot images (the seed images are larger because the single-shot images can have replicas originating from outside their FOV).

The CNN architecture is designed to match the physical parameters of the system and to process the data efficiently. The two inputs are concatenated at the very start. This is because the CMOS data alone cannot provide insight into the lifetime. Therefore, feature extraction layers are superfluous for the CMOS. Similarly, the CMOS constrains the information content of the iCCD.

After concatenation, 256 dilated convolutional kernels are applied, without zero-padding the inputs. Each kernel’s size is (11,1), due to the assumption of 6 replicas, centering the kernel on the 0th replica, with a dilation rate of 10 to match the pixel separation of replicas, creating a sparse receptive field of 101 pixels.

After the first convolutional layer, a second convolutional layer that outputs 64 feature maps is used and then a third that outputs 16 feature maps. The kernel sizes are still (11,1) with dilation rates of 10. These first three convolutional layers perform what amounts to blind deconvolution, doing much of the processing. Finally, 3 × 3 kernels output the final lifetime map. These 3 × 3 kernels help make the predictions smooth in a local 3 × 3 neighborhood, informing each pixel of the lifetime of its neighbors. This is equivalent to a sliding window binning strategy, except that the window does not use a simple sum, but rather a learned kernel. We find that this reduces image artifacts that might be present if each pixel were treated independently. The output feature map contains the lifetime prediction. We also experimented with predicting lifetime from the iCCD image only, discarding the CMOS image; *SI Appendix* for details.

The network is trained on 7,500 image triplets, validated on 1,250 and tested on 1,250. The training loss we used was mean squared error (MSE) weighted by intensity:

[10] $$\begin{array}{c} L=\frac{1}{N\times M}\sum_{i}\sum_{j}q_{i,j}{\left(\hat{\tau_{i,j}}-\tau_{i,j}\right)}^{2}, \end{array}$$

where N and M are the height and width of the ROI, q is the CMOS field of view corresponding to the lifetime label *τ*, and $\hat{\tau }$ is the predicted lifetime map. The motivation for scaling MSE is based on the variance of an unbiased lifetime predictor, which is inversely proportional to the total photon count. When measured photon counts are higher, SNR is higher too, and lifetime prediction can be expected to have lower variance; thus, any prediction error has a large significance since it cannot be attributed to a poor SNR and instead indicates poor fitting.

In summary, there are 3 physics-inspired aspects of this neural network. First, the usage of dilated kernels in our convolutional layers mimics the causal relationship between the lifetime prediction at a given pixel and the iCCD pixels above and below this target pixel, as described earlier. Second, the final layer is formed of 3 × 3 kernels, paralleling the sliding window binning strategy commonly used for increasing SNR in noisy measurement. Thirdly, our intensity-weighted mean squared error training loss simulates the variance of an unbiased lifetime predictor, which is inversely proportional to total fluorescent intensity due to the SNR of Poisson distributed photons from the sample (28).

Backpropagation was implemented via adaptive momentum (Adam), using an exponentially decaying learning rate for rapid initial training and eventual fine-tuning. The CNN was trained for 200 epochs, taking ≈ 6 h. Data generation/preprocessing and training/prediction were performed using an Intel(R) i9-9820X CPU and NVIDIA GeForce RTX 2080 Ti, respectively.

Both the preprocessing and CNN are computationally lightweight; for a single input, without special optimization, our code has a wall time of 21.6 ± 0.13 ms and 71.1 ± 6.5 ms, respectively, on the same computer. Hence, the processing pipeline can be run in real-time in parallel with a ≤10-Hz measurement.

### Uncertainty Analysis.

We analyze the improvement in the theoretical uncertainty of the lifetime prediction for general lifetimes. For this, we use a Bayesian prediction model.

Consider a signal of lifetime *τ*, creating an integrated CMOS measurement *q* and a series of noisy iCCD measurements $\hat{s}=\left({\hat{s}}_{1},{\hat{s}}_{2},...,{\hat{s}}_{3}\right)$. Here, ${\hat{s}}_{n}$ is the noisy iCCD signal in the n-th replica. We assume that the CMOS has an SNR far better than the iCCD camera; therefore, we ignore the CMOS in our noise calculations. Hence, we assume that the uncertainties in lifetime predictions originate from the iCCD only and the probability density function of our predictions likewise.

Continuing Eq. **3**, Bayes’ theorem states that

[11] $$\begin{array}{cc} p(\tau |\hat{s}) & =\frac{p\left(\hat{s}|\tau \right)p\left(\tau \right)}{p(\hat{s})}\\ & =\frac{p\left(\hat{s}|\tau \right)p\left(\tau \right)}{\int_{\tau }p\left(\hat{s}|\tau \right)p\left(\tau \right)d\tau }. \end{array}$$

We do not aim to make prior assumptions on *p*(*τ*) as we wish the system to be unbiased; however, for the sake of numerical calculation, we limit it between 0.02 and 20 ns. In this equation, $p(\hat{s}|\tau )$ is simply dependent on our noise model (for fixed total signal intensity), while the denominator can be numerically estimated by discretizing the integral.

Let us denote the variable denoting replica *i*’s iCCD signal as *S*_*i*_, with a value $\hat{s_{i}}$ in a particular measurement. *S*_*i*_ is distributed as

$$\begin{array}{c} S_{i}∼s_{i}\pm n. \end{array}$$

We expand Eq. **1** for *s*_*i*_ as

[12] $$\begin{array}{c} s_{i}=\int_{t}M\left(t\right)D_{\mathrm{QE}}P\left(t\right)dt, \end{array}$$

where *M* is the iCCD gain, *D*_*QE*_ is the detector quantum efficiency, and *P*(*t*) is the number of photons falling on the pixel at time t and is dependent on *τ*; finally, n is the noise (29). Eq. **1** describes the signal in our particular case as a function of lifetime *τ*.

The iCCD amplifies not only the Poissonian (signal) noise but also dark current noise and clock-induced charge (spurious) noise. Therefore, the noise, n, is

[13] $$\begin{array}{cc} n & ∼\sqrt{\sigma_{\mathrm{readout}}^{2}+F^{2}\int_{t}M^{2}\left(t\right)(\sigma_{\mathrm{signal}}^{2}\left(t\right)+\sigma_{\mathrm{dark}}^{2}+\sigma_{\mathrm{clc}}^{2})dt}\\ & \sigma_{\mathrm{signal}}^{2}\left(t\right)=D_{\mathrm{QE}}\cdot P\left(t\right), \end{array}$$

where F is the noise factor of the amplification process itself. Our Andor iStar 334T uses an 18x-73 intensifier, with quantum efficiency *D*_*QE*_ of around 20% at emission above 500 nm, dark current noise of 0.03 *e*^−^/*s* after amplification, readout noise 8 *e*^−^/*s*, and clock induced charge noise 0 *e*^−^/*s* (30). We combine Eqs. **12** and **13** as

[14] $$\begin{array}{c} S_{i}∼N\left(s_{i},n\right). \end{array}$$

We can then approximate the conditional probability density function $p(Si={\hat{s}}_{i}|\tau )$. We equate our noisy measurement $\hat{s}$ with a noisy lifetime prediction $\hat{\tau }$, giving us $p({\hat{\tau }}_{i}|\tau )$. We then multiply the *p*(*τ*) (uniform over [0.02,20] ns) and normalize by integrating over *τ* to obtain $p(\tau |{\hat{\tau }}_{i})$ as in Eq. **11**

Finally, from $p(\tau |\hat{\tau_{i}}))$, we estimate $p(\tau |\hat{\tau })$. For this, we assume that $\hat{\tau_{i}}$ are measurements of the same underlying variable, which is correct for our given noise model. Therefore,

[15] $$\begin{array}{c} \begin{array}{cc} p(\tau |\hat{\tau }) & =p\left(\tau |\hat{\tau_{0}},\hat{\tau_{1}},...,\hat{\tau_{n}}\right)=\prod_{i}p\left(\tau |\hat{\tau_{i}}\right). \end{array} \end{array}$$

This probability distribution provides the uncertainty (SD) of estimated lifetime $\hat{\tau }$ for some noisy measurement $\hat{s}$. This uncertainty is just a lower bound, as our assumptions of a thermal noise-free system reduce the noise of our estimates compared to a physical system. *SI Appendix* for a visualization of our uncertainty analysis.

## Supplementary Material

Appendix 01 (PDF)

## Data Availability

Raw images, synthetic training data (numpy arrays) for training a neural network, code required to process the data and produce the results in the paper, figures in svg and pdf formats. Data have been deposited in Single-shot time-folded fluorescence lifetime imaging (https://doi.org/10.5525/gla.researchdata.1380) (31).
