# Supplementary material for: Single-shot time-folded fluorescence lifetime imaging
Source: Proc Natl Acad Sci U S A. 2023 Apr 12;120(16):e2214617120. doi: 10.1073/pnas.2214617120 (PMC10120087; doi:10.1073/pnas.2214617120)
Supplement: Supplementary file 1 — Appendix 01 (PDF) [file pnas.2214617120.sapp.pdf]

Supporting Information for

Single-shot time-folded fluorescence lifetime imaging

V. Kapitany, V. Zickus, A. Fatima, G. Carles and D. Faccio

Valentin Kapitany
E-mail: v.kapitany.1@research.gla.ac.uk

This PDF file includes:

Supporting text
Figs. S1 to S7

Supporting Information Text

iCCD gate details

Fig. S1 shows the full iCCD gate gain profile with reference decay profile of simulated 1, 4 and 8 ns lifetime samples.

Replica angle and separation

Replicas created by the cavity are repeated copies of the field of view. As such, they can be seen on the 2D Fourier transform (2D-FT) of the iCCD image. To obtain good visibility, it is recommended to first subtract the mean of the iCCD from it, then Fourier transform it, then enhance the contrast locally, plot on a logarithmic scale, and finally crop the centre of the 2D-FT. In this enhanced 2D-FT, vertical replicas show up as clearly visible horizontal lines/bands above and below the main frequency cluster. The angle of these lines matches the angle of the replication direction. Furthermore, their offset from the centre is inversely proportional to the spatial offset between successive replicas, y (the farther apart the replicas, the nearer the lines to the centre).

Fig. S2 demonstrates the use of the 2D-FT for finding replica angle and separation. Fig. S3(a) shows a sample whose replicas are tilted by $\sim 3^\circ$ and the corresponding reconstructions; we note that some vertical artefacts appear. Fig. S3(b) shows the same sample, computationally rotated so that replicas are sheared along the vertical axis, and corresponding reconstructions. Artefacts are suppressed. Fig. S4 shows the same for a sample whose replica shear is tilted by $\sim 5^\circ$ from vertical.

Bead validation

Fig. S5 shows validation data measured by the FLIMera system quoted in the main text for our $2\mu m$ and 4 μm bead samples. The samples were imaged using a widefield TCSPC SPAD-array (FLIMera) and were fitted using maximum likelihood evaluation-based IRF deconvolution. Fig. S5 (a) shows the $2\mu m$ samples, while Fig. S5 (b) shows the $4\mu m$ samples. For both cases, we show (i) a global lifetime fit, (ii) a histogram of individual pixel fits (iii) the fluorescence intensity of the sample (iv) the sample's fluorescence lifetime, fitted pixel-wise.

Uncertainty analysis visualisation

We use Bayes' theorem in our uncertainty analysis, as:

$$\begin{aligned} p(\tau|\hat{s}) &= \frac{p(\hat{s}|\tau)p(\tau)}{p(\hat{s})} \\ &= \frac{p(\hat{s}|\tau)p(\tau)}{\int_\tau p(\hat{s}|\tau)p(\tau)d\tau} \end{aligned} \quad [1]$$

See Fig S6 (a-b), which show $p(\hat{s}_0|\tau)$ and $p(\hat{s}_3|\tau)$ for 5000 photons incident on the iCCD. These figures illustrate that very short lifetime signals decay mostly before the gate. This is particularly true for the first replica, where decay starts earliest and thus farthest from the iCCD gate, shown in Fig. S6 (a). Short lifetime signals are therefore not intensified and the detected e^- count is low; therefore, the signal-to-noise ratio (SNR) of the measurement will be poor. Conversely, long-lifetime signals are mostly uniformly intensified by the iCCD gate, giving similar signals. Fig. S6 (c-d) evaluate $p(\tau|\hat{\tau}_0)$ and $p(\tau|\hat{\tau}_3)$, respectively - we change variable by equating the noisy measurement $\hat{s}$ with $\hat{\tau}$. More simply: Fig. S6 (c) shows the probability density of the sample lifetime for some noisy 1st replica measurement, while Fig S6 (d) shows the same for the 3rd replica. The latter arrives later than the prior on the iCCD, due to the round-trip time of light in the optical cavity; consequently, the PDF $p(\tau|\hat{\tau}_3)$ is more sensitive to short lifetime signals than $p(\tau|\hat{\tau}_0)$. Conversely, $p(\tau|\hat{\tau}_3)$ is flatter than $p(\tau|\hat{\tau}_0)$ at high lifetimes as such samples give similar signals on the iCCD.

To understand this, we consider that if the lifetime is long, then the majority of the fluorescent decay lies in the quasi-homogeneous gain region of the iCCD (e.g. see the 8ns decay in Fig. S1, where the majority of the decay signal is in the ≈ 200 gain region). Then, most of the fluorescent decay intensity is uniformly amplified, ergo long-lifetime signals of identical total fluorescent intensity give similar readings on the iCCD. Since the ratios of the iCCD readings to the total intensity (CMOS reading), as well as the ratios of the various iCCD replicas to one another, contain information on the sample's lifetime, this means that long-lifetime samples are difficult to distinguish.

Fig. S6 (e) combines ($p(\tau|\hat{\tau}_0)$,$p(\tau|\hat{\tau}_1)$,...,$p(\tau|\hat{\tau}_5)$) into $p(\tau|\hat{\tau})$. Lastly, Fig. S6 (f) shows the [relative] standard deviation divided by the measured lifetime for a given true lifetime. We can see the effect of low measurement SNR on low lifetimes, increasing their relative uncertainty. We can also see the effect of degeneracy (poor distinguishability) between the measurements of high lifetime signals, causing their relative error to grow as well. The medium-long lifetime region between $\approx 0.3 - 3$ ns is where our detector works best.

Lifetime prediction without the CMOS

Close examination of our forward model shows that the relative intensities of the various replicas observed by the iCCD carry enough information to reconstruct fluorescence lifetime even in the absence of the CMOS camera. This is because the various iCCD replicas act like distinct time-gates.

However, we note that the reconstruction quality suffers greatly if the CMOS is omitted. We performed checks on the bead dataset shown in Fig. 3 of the main text, re-training our neural network architecture to output fluorescence lifetime without the CMOS input branch. Fig. S7 (c) and (e) show reconstruction results with and without the CMOS. Inclusion of the CMOS maintains contrast between the two beads much more consistently)

Fundamentally, the CMOS greatly constrains both the inverse retrieval and the CNN. Indeed, much of the information used to confine our methods arises from the ratio between the high-fidelity intensity measured on the CMOS and the intensity measured by the iCCD. Therefore, our work focuses on lifetime prediction with both a CMOS and the time-gated iCCD.

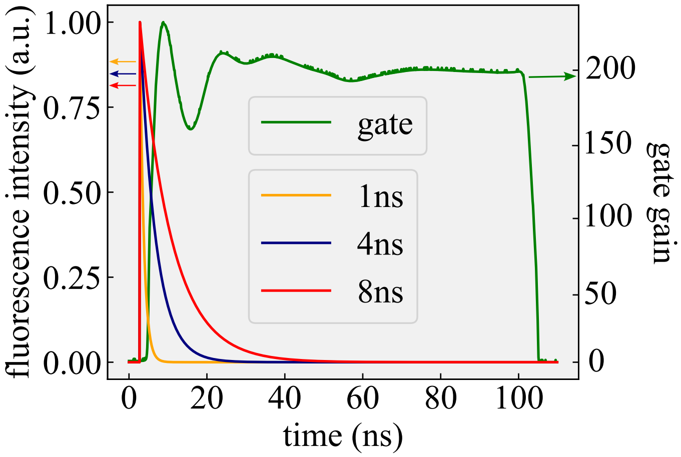

Fig. S1. We show the full gate gain profile, along with the first replicas of a 1ns, 4ns and 8ns FLIM sample. To increase the speed of calculations, our forward model simulates the decay (and gate) until the first 40ns, beyond which the signal is negligible even for high lifetimes.

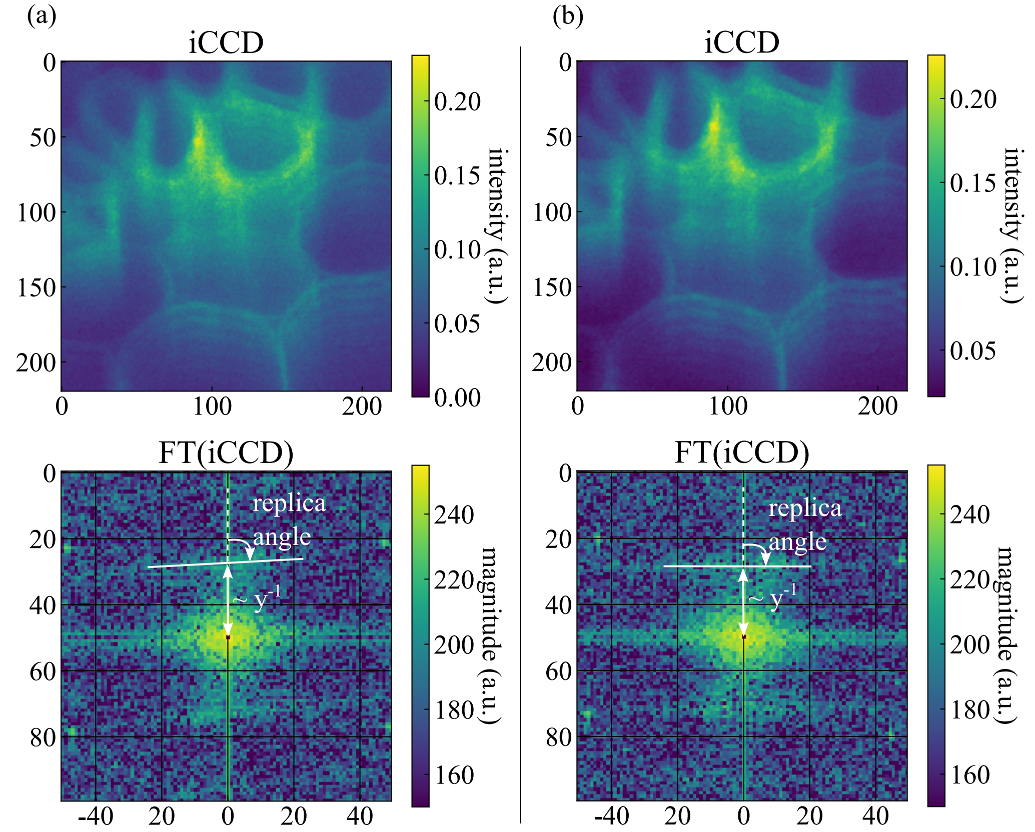

Fig. S2. We demonstrate the use of the 2D Fourier transform for finding replica angle and separation. **(a)** An iCCD image is shown; by eye, the replicas look approximately vertical. However, the 2D-FT shows the replicas as bands above (and via aliasing, below) the central frequencies. The angle swept by this band denotes measures the replication angle of the sample on the iCCD. Its offset from 0 is inversely proportional to the replica separation y. **(b)** A properly aligned image (with vertical replicas) has horizontal bands.

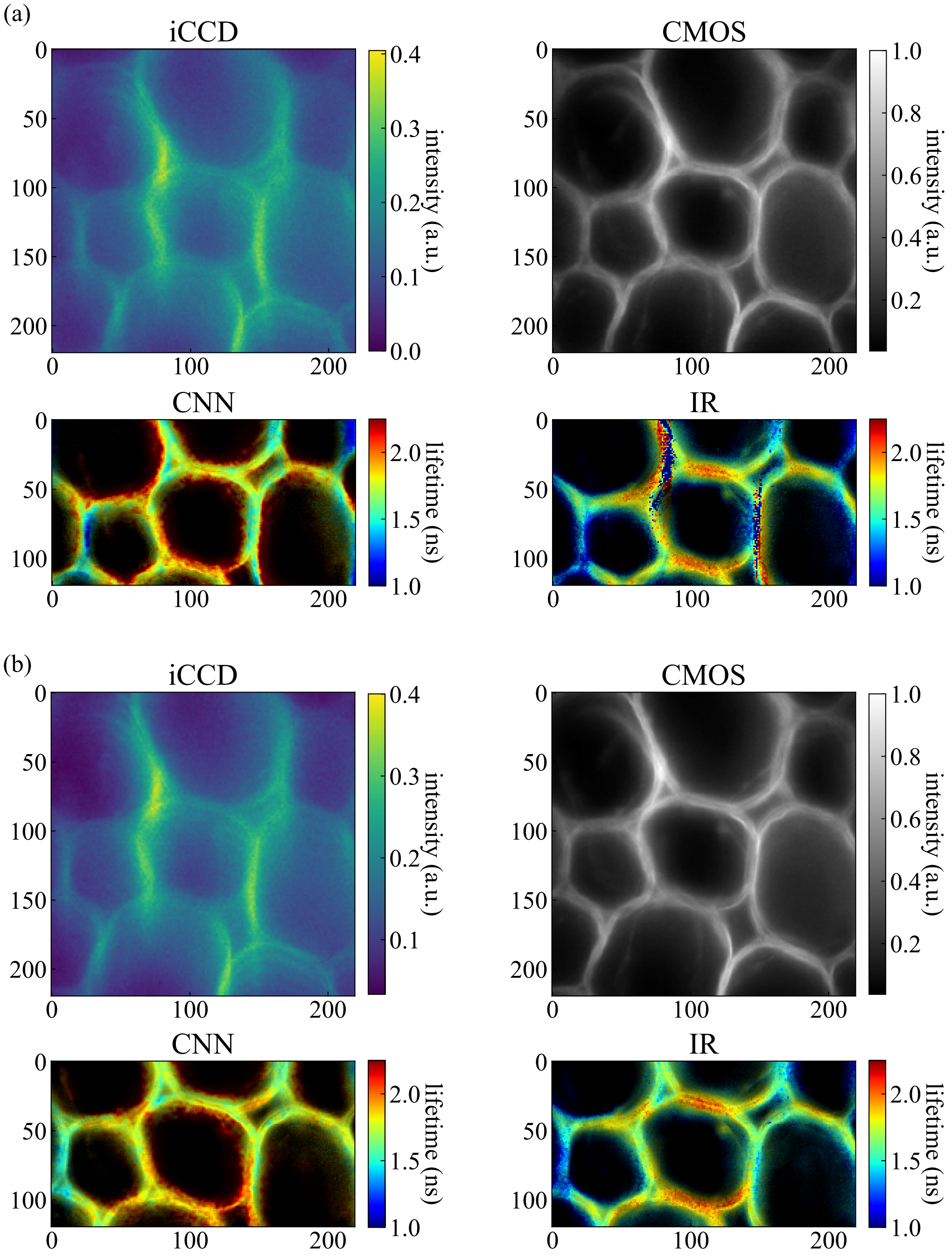

Fig. S3. **(a)** Data with replica shear tilted $\sim 3^{\circ}$ from vertical, and corresponding CNN and IR reconstructions. **(b)** The same sample with replicas sheared along the vertical axis, and corresponding reconstructions. Artefacts are suppressed.

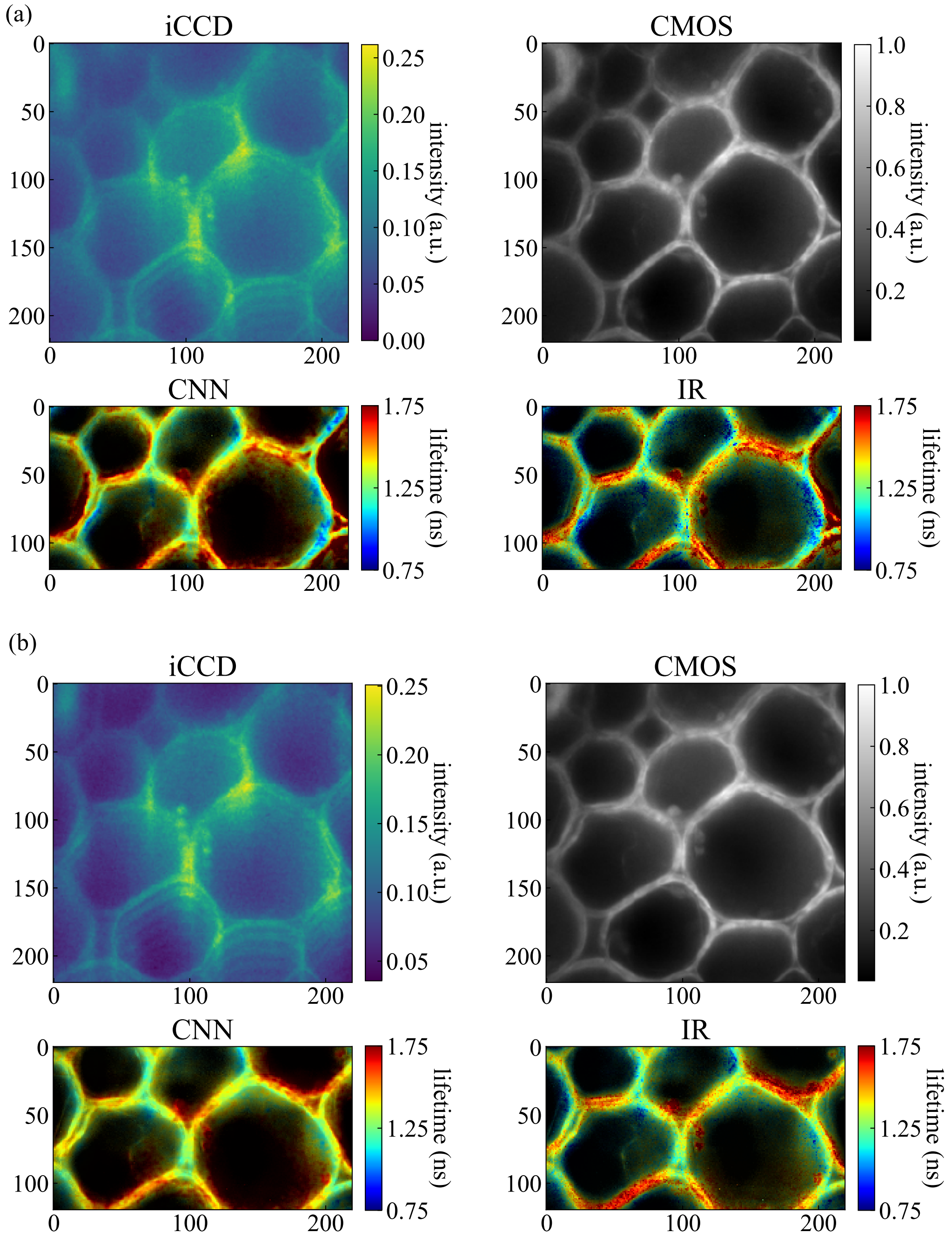

Fig. S4. **(a)** Data with replicas tilted $\sim 5^{\circ}$ from vertical, and corresponding CNN and IR lifetime estimates. **(b)** Data with replicas sheared along the vertical axis, and corresponding reconstructions.

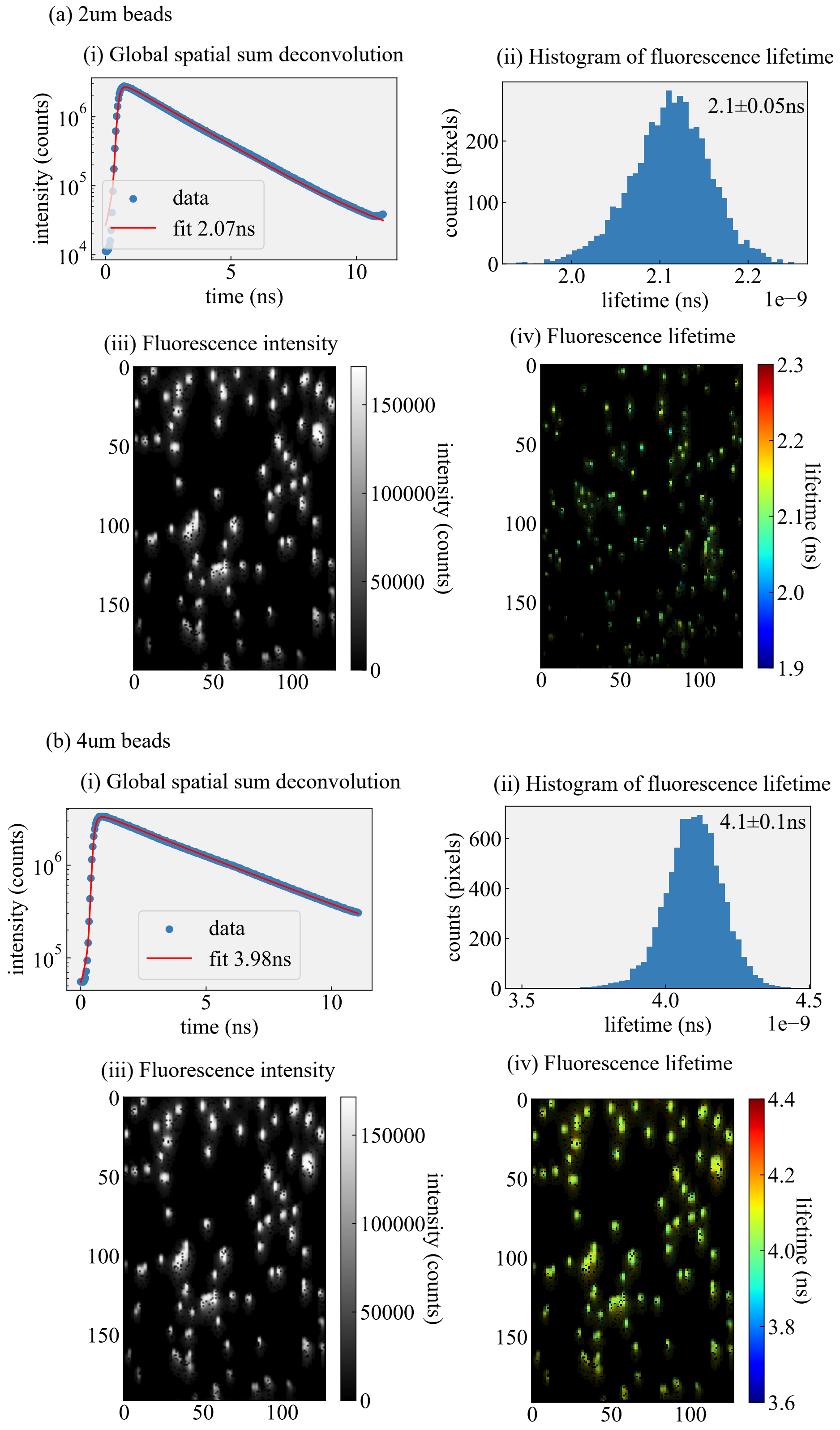

Fig. S5. We show validation of the $2\mu m$ and $4\mu m$ bead lifetimes, acquired with the FLIMera SPAD system. **(a)** Here we show data from the $2\mu m$ bead sample. **(i)** Displays the fit of the globally spatially summed SPAD datacube, with a lifetime of 2.07ns obtained via maximum likelihood evaluation (MLE) deconvolution. **(ii)** Shows a histogram of the lifetime values obtained when each pixel of the SPAD sample is fitted independently; we obtain a lifetime distribution of 2.1 ± 0.05ns **(iii)** shows the fluorescence intensity and **(iv)** the fluorescence lifetime. **(b)** Shows the same data for the $4\mu m$ beads, with a **(i)** global lifetime fit of 3.98ns and a **(ii)** pixelwise fit of 4.1 ± 0.1ns.

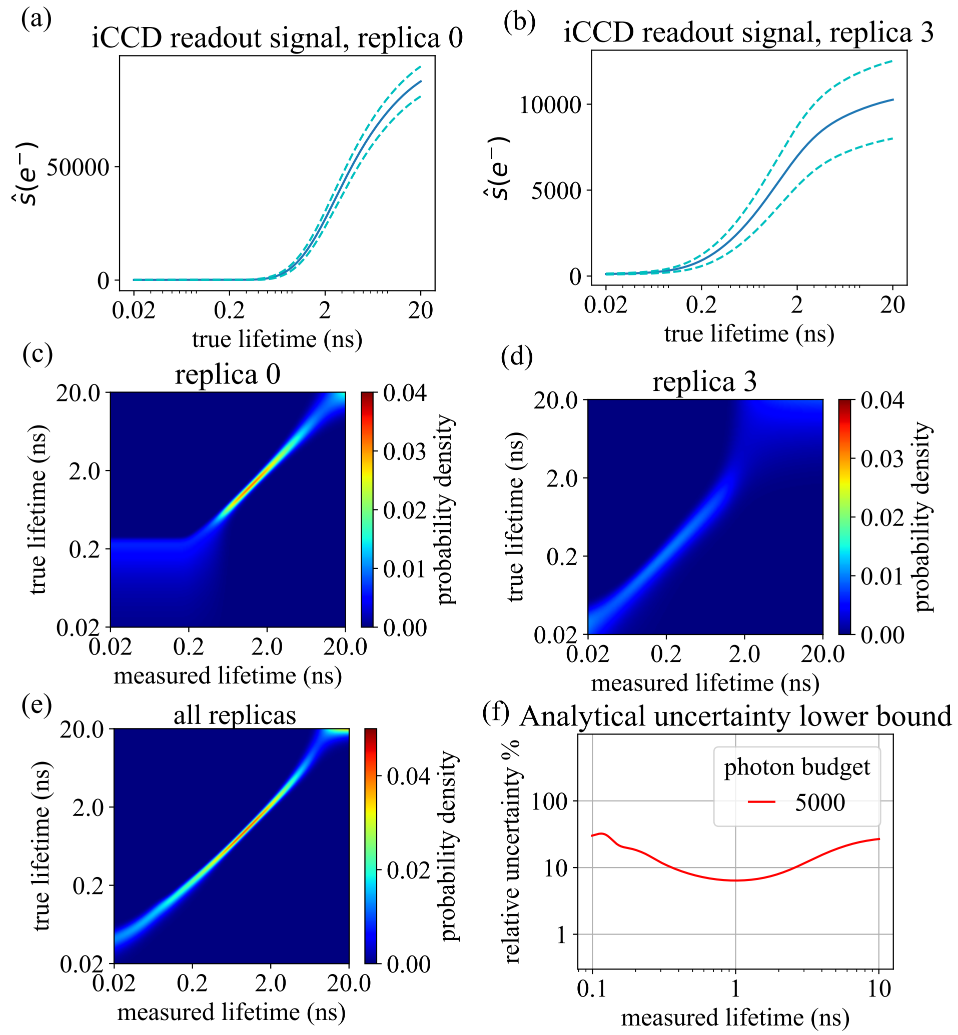

Fig. S6. Here we illustrate our method of estimating prediction uncertainty, assuming the 5000 photons arrive on the iCCD and a noise-free CMOS. **(a)** Shows the mean and standard deviation bounds of the expected electron count for a sample of a given true lifetime. The values shown are after intensification and noise, for the 0-th replica; **(b)** shows the same for the 3-rd replica. **(c)** Evaluates the probability density of the true (noise free) sample lifetime given the measured (noisy) sample for replica 0. The measured (noisy) sample lifetime is equivalent to the noisy electron counts in (a), whilst the true (noise free) lifetime is equivalent to (a)'s x-axis. **(d)** Shows the same for replica 3. **(e)** Shows the combined PDF of all replicas for a given sample, and is the [re-normalised] product of the PDFs of the various replicas. **(f)** Finally, we show relative uncertainty as a function of (true) lifetime. In other words, we evaluate the standard deviation in measured lifetime for fixed true lifetime (to get absolute uncertainty in ns) and divide by the true lifetime (to get relative uncertainty, unitless).

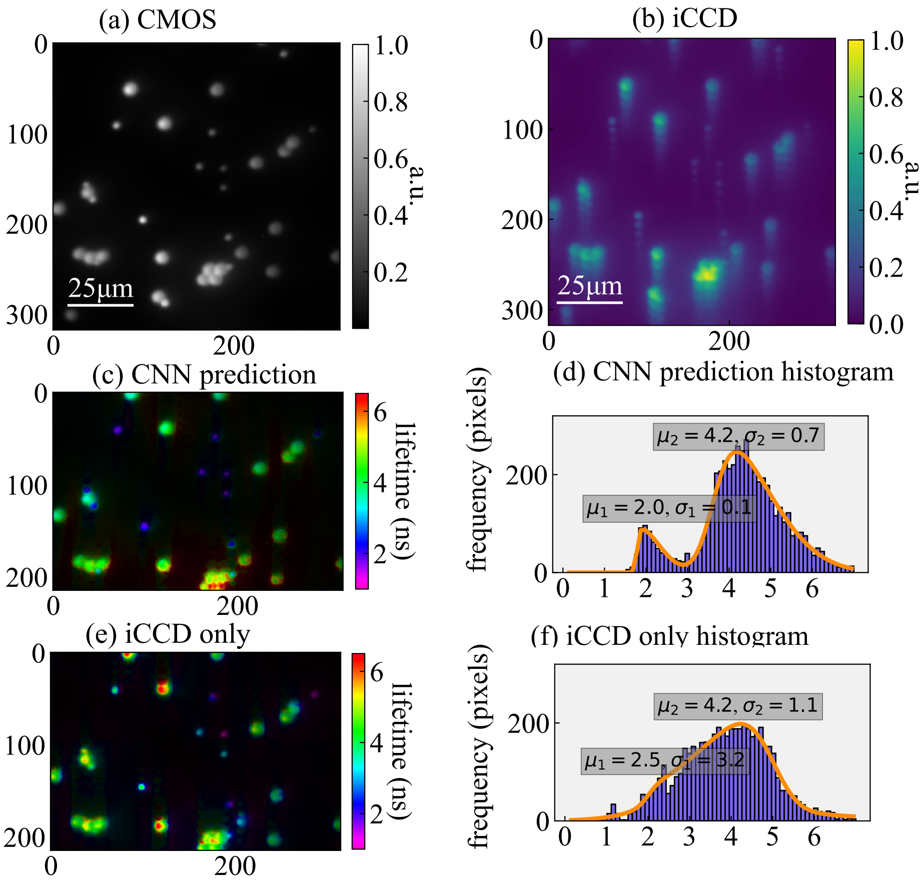

Fig. S7. Lifetime reconstructions of **(a-b)** our bead sample from Fig. 3 of the main text using a CNN, with and without the CMOS camera. The smaller beads are expected to show a $2ns$ lifetime while the larger ones are expected to have a lifetime of $4ns$. **(c-d)** The CNN which is given the CMOS and the iCCD produces a much more faithful lifetime reconstruction than the **(e-f)** CNN which only sees the iCCD image.
